# Factors affecting academic burnout of nursing students according to clinical practice experience

**DOI:** 10.1186/s12909-022-03422-7

**Published:** 2022-05-06

**Authors:** Eunhee Hwang, Jeonghyun Kim

**Affiliations:** 1 Department of Nursing, WonKwang University, 460 Iksandae-ro, Iksan, Jeonbuk 54538 Republic of Korea; 2 College of Nursing, Catholic University of Pusan, 57 Oryundae-ro, Geumjeong-gu, Busan, 46252 Republic of Korea

**Keywords:** Nursing students, Burnout, Stress, Depression, Anxiety

## Abstract

**Background:**

Academic burnout has a negative effect on learning outcomes of nursing students. Factors affecting academic burnout may differ depending on whether or not they have experience in clinical practice and identifying these differences would be necessary to seek for strategies to lower academic burnout of nursing students. This study aimed to determine the effects of stress, depression, and anxiety on academic burnout according to the clinical practice experience of nursing students.

**Methods:**

Data were collected from 171 female nursing students in South Korea. Self-report questionnaires from 83 participants without clinical practice experience and 88 with clinical practice experience were analyzed using descriptive statistics, χ2-tests, analysis of variance, t-test, Spearman correlation coefficient, and stepwise multiple regression.

**Results:**

Academic burnout was positively correlated to stress (*r* = .52, *p* < .001), anxiety (*r* = .50, *p* < .001) and depression (*r* = .44, *p* < .001). In those students with no clinical practice experience, anxiety and depression explained for academic burnout by 44%, and those students with clinical practice experience, stress and major satisfaction explained for 33% of academic burnout.

**Conclusions:**

Universities and clinical institutions should establish a cooperative system to reduce stress, depression and anxiety and increase major satisfaction.

## Introduction

Burnout is an important issue in work life as well as studies of students [1]. Nursing students experience high levels of stress due to significant workload, relatively inflexible curriculum, competitive atmosphere between peers, and preparation for nurse license national exam [2], which inevitably lead to burnout. As a result, burnout causes psychological and physical problems (weakness, insomnia), emotional problems (anxiety, depression), attitude problems (hostility, apathy, distrust), and behavioral problems (aggression, nervousness) [3].

Academic burnout is a psychological symptom caused by excessive academic burden and continued academic stress. Academic burnout consists of emotional exhaustion, apathy, and incompetence. Emotional exhaustion refers to feeling exhausted because of study demands. Apathy is having a cynical and detached attitude toward one’s study, and incompetence means to feeling incompetent as a student [4]. Academic burnout in nursing students leads to loss of confidence and acts as a negative factor that causes psychological withdrawal and frustration in their studies [5]. Additionally, academic burnout is the most significant factor predicting psychological well-being [6] and is a factor that interferes with job preparation, transfer of professional role, and socialization [7]. Therefore, educational strategies to reduce burnout are essential.

According to previous studies, the variables that affect academic burnout include personal intrinsic factors such as health status, interpersonal relationships, anxiety, depression, psychological stress, and self-efficacy, environmental factors such as relationships with parents, and friends, and major-related factors such as nursing professionalism or major satisfaction [8–13]. Nursing studies are associated with high levels of stress due to the nature of the curriculum, and nursing students often experience depression and anxiety. In a study on nursing students in Hong Kong, 35.8% of the students experienced depression while 37.3% and 41.1% experienced anxiety and stress, respectively [14]. In Canada, nursing students had higher levels of depression, anxiety, and stress compared to other students [15], and similarly in Korea, nursing students experienced higher levels of depression, anxiety, and stress compared to other major students [16, 17]. Additionally, compared to other major students, medical school and nursing students experienced higher levels of burnout due to the complex curriculum and pressure for professional performance [18]. Altogether, these evidence show that nursing students frequently experience psychological and emotional problems such as academic exhaustion, stress, depression, and anxiety during their four years of completing their degree.

In Korea, the undergraduate program for nursing is four-year-long with a total of eight semesters. In the first year, nursing students adapt to university life and understand their major, and in the second year, students complete the basic courses for their major and gradually increase their understanding through basic nursing practice in classrooms and labs. In the third year, nursing students obtain their first clinical experience, and in the fourth year, the students grow interest in employment, national exam preparation, and graduation along with clinical practice. The experience of clinical practice is the key aspect of nursing curriculum and serves as the basis for becoming professional nurses after graduation.

As such, nursing students face unique characteristics of the curriculum every year and changes in different educational environments. In this process, the students may experience burden and stress, which lead difficulties in adapting. In particular, Korean nursing students must complete more than 1,000 h of clinical practice during their third and fourth years in order to graduate. Main criticism during the clinical practice of nursing students include observation-oriented practice, unsystematic educational performance evaluation, and lack of feedback on evaluation results [19]. As a result, nursing students experience stress from clinical practice, and such stress and dissatisfaction with clinical practice affect academic burnout [20–22]. Therefore, factors affecting academic burnout may differ depending on whether or not they have experience in clinical practice and identifying these differences would be the first step to seek for strategies to lower academic burnout of nursing students.

In this study, we aimed to evaluate the levels of academic burnout of nursing students and identify factors affecting academic burnout according to clinical practice experience. The detailed aims of this study were as follows.

1. Assess the difference in stress, depression, anxiety, and academic burnout of nursing students according to clinical practice experience
2. Assess difference in academic burnout according to general characteristics of nursing students
3. Investigate the correlation between stress, depression, anxiety, and academic burnout according to clinical practice experience
4. Identify factors affecting academic burnout of nursing students according to clinical practice experience

## Methods

### Research design

This was a descriptive cross-sectional study to determine the effects of stress, depression, and anxiety on academic burnout according to the clinical practice experience of nursing students.

### Participants

The participants of this study were female nursing students in first to fourth years enrolled in the Department of Nursing. First- and second-year nursing students without clinical practice experience were considered as those without clinical practice experience. Third- and fourth-year students were considered as those with clinical practice experience. The questionnaire was conducted in the second half of the semester. A total of 180 participants, including 90 participants each with and without clinical practice experience, were surveyed. Questionnaires from 171 participants (83 participants without clinical practice experience and 88 with clinical practice experience), excluding nine questionnaires with insincere responses, were analyzed. G Power program 3.1.9.2 was used to calculate the number of participants. Effect size of 0.30 by referring to previous studies related to clinical practice stress and depression of nursing students [23, 24], power of 0.80, significance level of 0.05, and 14 variables were set to conduct F-test, and the required number of participants was 74 for each group. The number of participants in each group satisfied the criteria.

### Research tools

#### Stress

Stress is defined that expresses a state of psychological conflict which occurs when a risk from the outside exceeds the level of the ability to react or puts a risk on the resources of each individual that is kept constant [25]. Stress was evaluated using an 18-item tool developed by Jang[ [25] based on the Psychosocial Wellbeing Index (PWI), a social psychological health tool that measures the level of general mental health. The tool was evaluated on a 4-point Likert scale with 0 point for 'every time', 1 point for 'often', 2 points for 'rarely', and 3 points for 'never'. Negative items were inversely converted. A higher score indicated higher stress, and Cronbach's α was 0.87 in the study by Jang [25] and 0.89 in our study.

#### Depression

Depression refers affective symptoms including depressed mood, feelings of guilt and worthlessness, feelings of helplessness and hopelessness, psychomotor retardation, loss of appetite, and sleep disturbance [26]. Depression was evaluated using 20-item scale validated by Chen et al. [27]. This scale was based on Center for Epidemiologic Studies Depression developed by Radloff [26], which evaluated the frequency of depression symptoms in the previous week. The scale was evaluated on a 4-point Likert scale with a higher score indicating greater depression. Cronbach's α was 0.85 in the study by Radloff [26] and, 0.86 in our study.

#### Anxiety

Anxiety is defined as a psychological emotional state or reaction including unpleasant feeling of tension, apprehension, nervousness and worry, and activation of the automonic nervous system [28]. Anxiety was evaluated using a standardized Korean version of Spielberger's State-Trait Anxiety inventory [28] by Kim & Shin [29]. The scale consisted of 20 items evaluated on a 4-point Likert scale. A higher score indicated higher levels of anxiety. Cronbach's α was 0.87 in the study by Kim & Shin [29] and 0.92 in our study.

#### Academic burnout

Academic burnout refers a psychological symptom caused by excessive academic burden and continued academic stress [4]. Maslach Burnout Inventory-Student Survey (MBI-SS) developed by Shaufeil et al. [4] for college students and validated for Korean students by Shin et al. [30] was used to evaluate academic burnout. The tool consisted of 15 items in total with three sub-factors (exhaustion, apathy, and incompetence). The items were evaluated on a 5-point Likert scale. Incompetence was measured by inverse scoring, and a higher score indicated higher tendency to burn out. Cronbach's α was 0.82 ~ 0.86 in the study by Shin et al. [30] and 0.82 in our study.

### Data analysis

SPSS/WIN 25.0 program was used to analyze the collected data. Compliance to normal distribution was assessed via descriptive statistics and the Shapiro–Wilk test. Data were not normally distributed; therefore, non-parametric statistical tests were used to analyze study data. T-test and χ2-test were conducted to compare differences in general characteristics according to clinical practice experience, and t-test and Mann–Whitney U test were conducted to assess differences in stress, depression, anxiety, and academic burnout. T-test, analysis of variance (ANOVA), and Tukey post-hoc test were conducted to evaluate differences in academic burnout according to the general characteristics, and Spearman correlation coefficient analysis was performed to assess the correlation between stress, depression, anxiety, and burnout. To identify factors influencing academic burnout, stepwise multiple regression analysis was performed with entry level below 0.05 and exit level above 0.1.

### Ethical considerations

This study was approved by the Institutional Review Board (IRB) of Wonkwang university for ethical considerations (IRB approval number: WKIRB-201902-SB-020). The participants were students of the researcher's affiliated institution and were considered vulnerable. Thus, to prevent compulsory participation and ensure voluntary participation, a trained research assistant provided explanations of the study and asked for voluntary consent to the participants. In addition, the participants were explained that there would be no penalties on grades or other disadvantages for refusing to participate in the study. The purpose of the study and anonymity and confidentiality of the study were explained to the participants, and the questionnaire was distributed only to those who voluntarily agreed to participate and provided a written consent form. The questionnaire required 10 to 15 min for completion.

## Results

### General characteristics of the participants

A total of 171 participants were included in the final analysis, and 83 (48.5%) and 88 (51.5%) participants had and did not have clinical practice experience, respectively. Approximately 60.2% of the participants were not religious and 74.3% did not live with their parents. About 52.0% of the participants were introverted with the remaining 48.0% of the participants being extrovert, and 73.1% of the participants responded that they had good interpersonal relationships. The most common subjective health was moderate at 53.8%, and 72.5% of the participants did not exercise regularly. Approximately 68.4% and 69.6% of the participants were satisfied with their major and professors, respectively, and 87.1% of the participants showed had high implications for nursing.

The mean age of the participants with clinical practice experience was significantly higher than that of those without clinical experience (t = 8.40, *p* < 0.001). There was no significant difference in other characteristics according to clinical practice experience (Table 1).

**Table 1:** General characteristics of the participants (*n* = 171)

Characters	Categories	Total	Practice experience		χ^2^ or t	*p*
			No (*n* = 83)	Yes (*n* = 88)		
		n (%)	n (%) / Mean ± SD			
Age(year)			19.71 ± 1.73	21.91 ± 1.69	8.40	< .001
Religion	Yes	68(39.8)	34(50.0)	34(50.0)	0.10	.756
	No	103(60.2)	49(47.6)	54(52.4)		
Living with parents	Yes	44(25.7)	22(50.0)	22(50.0)	0.05	.822
	No	127(74.3)	61(48.0)	66(52.0)		
Character	Introvert	89(52.0)	43(48.3)	46(51.7)	0.00	.951
	Extrovert	82(48.0)	40(48.8)	42(51.2)		
Interpersonal relationship	Good	125(73.1)	60(48.0)	65(52.0)	0.05	.816
	Moderate	46(26.9)	23(50.0)	23(50.0)		
Subjective health status	Good	37(21.6)	23(62.2)	14(37.8)	4.18	.124
	Moderate	92(53.8)	39(42.4)	53(57.6)		
	Bad	42(24.6)	21(50.0)	21(50.0)		
Regular exercise	Yes	47(27.5)	26(53.3)	21(44.7)	1.19	.275
	No	124(72.5)	57(46.0)	67(54.0)		
Major satisfaction^a^	Good	117(68.4)	60(51.3)	57(48.7)		.094
	Moderate	49(28.7)	23(46.9)	26(53.1)		
	Bad	5(2.9)	0(0.0)	5(100.0)		
Satisfaction with professor^a^	Good	119(69.6)	64(53.8)	55(46.2)		.122
	Moderate	47(27.5)	17(36.2)	30(63.8)		
	Bad	5(2.9)	2(40.0)	3(60.0)		
Implications for Nursing^a^	High	149(87.1)	76(51.0)	73(49.0)		.269
	Moderate	15(8.8)	5(33.3)	10(66.7)		
	Low	7(4.1)	2(28.6)	5(71.4)		

^a^Fisher’s exact tests

### Differences in stress, depression, anxiety, and academic burnout according to clinical practice experience

The group without clinical practice experience had a higher level of stress (t = 2.65, *p* = 0.009) and depression (z = -2.00, *p* = 0.045) than those with clinical practice experience. The total scores for anxiety was higher in those without clinical practice; however, the difference was not statistically significant (Table 2).

**Table 2:** Level of stress, depression and anxiety according to clinical practice experience (*n* = 171)

Variables	range	Total score mean ± SD (Item mean ± SD)				
		Practice experience		t	*z*	*p*
		No (*n* = 83)	Yes (*n* = 88)			
Stress	0–54 (0–3)	19.93 ± 7.71 (1.11 ± 0.43)	16.88 ± 7.33 (0.94 ± 0.41)	2.65		.009
Depression^a^	0–60 (0–3)	13.65 ± 9.72 (0.68 ± 0.49)	10.94 ± 8.16 (0.55 ± 0.41)		-2.00	.045
Anxiety	20–80 (1–4)	41.34 ± 9.85 (2.07 ± 0.49)	39.33 ± 8.89 (1.97 ± 0.44)	1.40		.163

^a^Mann-Whitney U test

The total score of academic burnout was higher in the group without clinical practice experience, but the difference was not statistically significant. All of the subcategories, exhaustion, apathy, and incompetence, also showed no statistically significant difference between groups (Table 3).

**Table 3:** Level of academic burnout according to clinical practice experience (*n* = 171)

Variables	Practice experience		t	*p*
	No (*n* = 83)	Yes (*n* = 88)		
	Mean ± SD			
Academic burnout	2.72 ± 0.59	2.69 ± 0.53	0.44	.658
Exhaustion	3.17 ± 0.95	3.27 ± 0.90	0.67	.504
Apathy	2.19 ± 0.85	2.10 ± 0.79	0.72	.473
Incompetence	2.71 ± 0.62	2.59 ± 0.55	1.28	.203

### Differences in academic burnout according to general characteristics

The differences in academic burnout according to the general characteristics of the participants are shown in Table 4. Those who had good subjective health experienced lower academic burnout than those who responded to have bad subjective health (F = 4.65, *p* = 0.011). Additionally, academic burnout was lower in those with higher major satisfaction than in those with low satisfaction (F = 6.33, *p* = 0.002). Academic burnout was significantly lower in those who had high satisfaction with professors than those with low satisfactions (F = 6.83, *p* = 0.001) and lower in those with high implications for nursing than those with low implications (F = 5.66, *p* = 0.004).

**Table 4:** Differences of academic burnout according to general characteristics

Variables		Total (*N* = 171)		Practice experience			
				No (*n* = 83)		Yes (*n* = 88)	
		Mean ± SD	t, F(*p*) Tukey	Mean ± SD	t, F(*p*) Tukey	Mean ± SD	t, F(*p*) Tukey
Religion	Yes	2.68 ± 0.54	0.58(.566)	2.76 ± 0.58	0.74(.462)	2.61 ± 0.51	1.66(.101)
	No	2.74 ± 0.59		2.67 ± 0.62		2.80 ± 0.55	
Living with parents	Yes	2.59 ± 0.55	1.53(.129)	2.65 ± 0.51	0.67(.504)	2.53 ± 0.60	1.54(.128)
	No	2.74 ± 0.56		2.75 ± 0.62		2.74 ± 0.50	
Character	Introvert	2.78 ± 0.52	1.85(.066)	2.81 ± 0.57	1.36(.178)	2.75 ± 0.47	1.24(.217)
	Extrovert	2.62 ± 0.59		2.63 ± 0.60		2.61 ± 0.59	
Interpersonal relationship	Good	2.69 ± 0.59	0.48(.630)	2.72 ± 0.65	0.06(.955)	2.66 ± 0.54	0.64(.523)
	Moderate	2.74 ± 0.47		2.73 ± 0.41		2.75 ± 0.53	
Subjective health status	Good^a^	2.49 ± 0.72	4.65(.011) a < c	2.54 ± 0.72	2.48(.090)	2.43 ± 0.75	2.39(.098)
	Moderate^b^	2.71 ± 0.49		2.72 ± 0.48		2.70 ± 0.49	
	Bad^c^	2.87 ± 0.50		2.93 ± 0.59		2.82 ± 0.41	
Regular exercise	Yes	2.60 ± 0.54	1.45(.149)	2.56 ± 0.54	1.77(.081)	2.66 ± 0.55	0.22(.823)
	No	2.74 ± 0.57		2.80 ± 0.60		2.69 ± 0.53	
Major satisfaction	Good^a^	2.62 ± 0.56	6.33(.002) a < c	2.71 ± 0.62	0.11(.742)	2.53 ± 0.48	10.34(< .001) a < b,c
	Moderate^b^	2.83 ± 0.48		2.76 ± 0.53		2.90 ± 0.43	
	Bad^c^	3.36 ± 0.73		-		3.36 ± 0.73	
Satisfaction with professor	Good^a^	2.61 ± 0.52	6.83(.001) a < b	2.67 ± 0.53	1.42(.249)	2.53 ± 0.51	7.02(.002) a < b
	Moderate^b^	2.91 ± 0.46		2.90 ± 0.49		2.91 ± 0.46	
	Bad^c^	3.11 ± 1.37		3.07 ± 0.59		3.13 ± 0.72	
Implications for Nursing	High^a^	2.66 ± 0.55	5.66(.004) a < c	2.71 ± 0.60	1.36(.263)	2.60 ± 0.48	6.21(.003) a < c
	Moderate^b^	2.88 ± 0.54		2.67 ± 0.46		2.99 ± 0.57	
	Low^c^	3.31 ± 0.56		3.40 ± 0.00		3.28 ± 0.69	

*a,b,c subgroups classified based on the mean values of academic burnout

There were no significant differences in academic burnout according to general characteristics of participants without clinical practice experience.

In contrast, in participants with clinical practice experience, those who had high major satisfaction (F = 10.34, *p* < 0.001), high satisfaction with professors (F = 7.02, *p* = 0.002), and high implications for nursing (F = 6.21, *p* = 0.003) had significantly lower academic burnout score than those who had low major satisfaction, low satisfaction with professors, and low implications for nursing.

### Correlation between academic burnout and stress, depression, and anxiety according to clinical practice experience

The correlation between academic burnout and other variables was analyzed (Table 5). Academic burnout was positively correlated with stress (*r* = 0.52, *p* < 0.001), anxiety (*r* = 0.50, *p* < 0.001) and depression (*r* = 0.44, *p* < 0.001). Among the subcategories of academic burnout, exhaustion was highly correlated with anxiety (*r* = 0.45, *p* < 0.001). Apathy (*r* = 0.47, *p* < 0.001) and incompetence (*r* = 0.32, *p* < 0.001) showed higher correlation coefficient with stress.

**Table 5:** Correlation among stress, depression, anxiety, and academic burnout

	Total (*n* = 171)			No (*n* = 83)			Yes (*n* = 88)		
	Stress	Depression	Anxiety	Stress	Depression	Anxiety	Stress	Depression	Anxiety
Academic burnout	.52^**^	.44^**^	.50^**^	.52^**^	.48^**^	.52^**^	.54^**^	.42^**^	.49^**^
Exhaustion	.35^**^	.35^**^	.45^**^	.41^**^	.47^**^	.57^**^	.33^*^	.26^*^	.35^*^
Apathy	.47^**^	.30^**^	.34^**^	.41^**^	.28^*^	.29^*^	.53^**^	.32^*^	.41^**^
Incompetence	.32^**^	.31^**^	.30^**^	.27^*^	.26^*^	.26^*^	.35^*^	.35^*^	.32^*^

^*^ *p* < .01, ** *p* < .001

In those participants without clinical practice experience, academic burnout was correlated higher with stress and anxiety (*r* = 0.52, *p* < 0.001). Among the subcategories, exhaustion and apathy showed the highest correlation coefficient with anxiety and stress, respectively. The correlation coefficient between incompetence and other variables was 0.3 or less.

On the other hand, in those with clinical practice experience, academic burnout was correlated with stress (*r* = 0.54, *p* < 0.001), anxiety (*r* = 0.49, *p* < 0.001), and depression (*r* = 0.42, *p* < 0.001) in a decreasing order. The correlation coefficient between apathy and stress was the highest (*r* = 0.53, *p* < 0.001), and the other subcategory variables except exhaustion and depression also showed correlation coefficients of 0.3 or higher with stress, depression and anxiety.

### Factors affecting academic burnout according to clinical practice experience

Factors affecting academic burnout of nursing students are shown in Table 6. Subjective health, major satisfaction, satisfaction with professors, implications for nursing, stress, anxiety, and depression, which led to significant differences in academic burnout, were included in regression analysis. Among the general characteristics of the participants, subjective health, major satisfaction, satisfaction with professors, and implications for nursing were treated as dummy variables.

**Table 6:** Factors affecting academic burnout

Variables	Total (*n* = 171)					Practice experience									
						No (*n* = 83)					Yes (*n* = 88)				
	B	SE	β	t	*p*	B	SE	β	t	*p*	B	SE	β	t	*p*
constant	2.04	0.22		9.30	< .001	1.59	0.28		5.86	< .001	2.35	0.15		15.11	< .001
Anxiety	0.39	0.12	0.33	3.28	.001	0.02	0.01	0.38	2.64	.010					
Depression	0.02	0.01	0.25	2.51	.013	0.40	0.17	0.33	2.30	.024					
Satisfaction with professor (ref = moderate, bad)^a^	-0.16	0.08	-0.15	2.34	.020										
Implication for nursing (ref = moderate, low)^b^	-0.24	0.10	-0.13	2.08	.039										
Stress											0.03	0.01	0.45	4.96	< .001
Major satisfaction (ref = moderate, bad)^a^											-0.30	0.10	-0.27	2.90	.005
F(*p*)	26.67(< .001)					33.20(< .001)					22.06(< .001)				
R^2^	0.39					0.45					0.34				
adjusted R^2^	0.38					0.44					0.33				

^a^Dummy variable (1 = good, 0 = moderate, bad)

^b^Dummy variable (1 = high, 0 = moderate, low)

Durbin-Watson statistics value, which assess autocorrelation between error terms, was 2.03. The value was close to 2, suggesting that there is no autocorrelation between the error terms. The variance expansion factor ranged from 1.02 to 3.09, which was less than 10 and suggested there was no multicollinearity between independent variables. Thus, the basic assumptions for multiple regression analysis were satisfied.

Factors affecting academic burnout of nursing students were anxiety (β = 0.33, *p* = 0.001), depression (β = 0.25, *p* = 0.013), satisfaction with professors (β = -0.15, *p* = 0.020), and implications for nursing (β = -0.13, *p* = 0.039). In other words, those with higher anxiety and depression had greater academic burnout and those with good satisfaction with professors and high implications for nursing had lower academic burnout. When only the anxiety variable was initially input, the effect size was 0.32, but as implications for nursing, depression, and satisfaction with professors were added in sequence, the magnitude of the effect increased to 0.34 and 0.36, respectively, and the final explanatory power of four variables was 38% (F = 26.67, *p* < 0.001).

In those participants without clinical practice experience, Durbin-Watson statistics value was 1.82, suggesting no autocorrelation between the error terms. The variance expansion factor was 3.00, which was less than 10. Thus, there was no multicollinearity between independent variables, and the basic assumptions for regression analysis were satisfied. Academic burnout of nursing students without clinical practice experience was higher with greater anxiety (β = 0.38, *p* = 0.010) and depression (β = 0.33, *p* = 0.024). The effect size of depression in the model was 0.41, and the final explanatory power for academic burnout including anxiety was 44% (F = 33.20, *p* < 0.001).

In those participants with clinical practice experience, Durbin-Watson statistics value was 2.02, suggesting no autocorrelation between the error terms. The variance expansion factor was 1.10, which was less than 10. Thus, there was no multicollinearity between independent variables, and the basic assumptions for regression analysis were satisfied. Academic burnout of nursing students with clinical practice experience increased with higher levels of stress (β = 0.45, *p* < 0.001) and those with good major satisfaction had lower academic burnout (β = -0.27, *p* = 0.005). The effect size of stress was 0.27, and the effect size increased with the input of major satisfaction, so that the final explanatory power of the two variables was 33% (F = 22.06, *p* < 0.001).

## Discussion

This study aimed to evaluate the differences in academic burnout according to the clinical practice experience of nursing students and identify factors affecting academic burnout. In this discussion, we show the level of academic burnout of nursing students, present factors affecting academic burnout according to clinical practice experience, and suggest measures to reduce academic burnout in order to provide basic data for strategies to promote academic performance of nursing students.

The academic burnout score was 2.69 points in those without clinical practice experience and 2.72 points in those with clinical practice experience. There was no significant difference in academic burnout according to clinical practice experience. The academic burnout score observed in our study was lower than that measured in previous studies using the same tool. In first- and second-year nursing students, the academic burnout score was 2.84–2.90 in a previous study [31]. In another study, third- and fourth-year nursing students had an academic burnout score of 2.9–3.0. The lower academic burnout score in our study may be attributed to the differences in the time point at which academic burnout was assessed. Unlike in previous studies that measured academic burnout at the end of the semester, in our study, academic burnout was evaluated seven to eight weeks after the start of the semester. However, first- and second-year nursing students without clinical practice experience showed similar levels of academic burnout as third- and fourth-year nursing students with clinical practice experience. This suggests the importance of managing and preventing academic burnout throughout the whole four years of nursing curriculum.

Herein, first- and second-year students without clinical practice experience showed higher levels of stress compared to third- and fourth-year students. Stress is a key factor affecting academic burnout of nursing students [20, 21]. In previous students, the differences in stress levels according to the year of curriculum and clinical practice experience varied. Jung (2019) showed that the first-year students had the highest levels of stress [32]. On the other hand, An (2017) observed that third year students had higher levels of stress than first- and fourth-year students [22]. In another study by Moon (2018), there was no correlation between stress level and year of the curriculum [33]. Therefore, exploratory studies on stress-related factors of first- and second-year nursing students without clinical practice experience and changes in stress pattern according to the year of the curriculum would be required to improve the academic performance and successful adaptation to university life of nursing students.

Our data showed differences in academic burnout according to clinical practice experience. In those participants who had clinical practice experiences, academic burnout differed according to major satisfaction, satisfaction with professors, and implications for nursing. In contrast, in those without clinical experience, there was no difference in academic burnout according to general characteristics. These findings suggest that clinical practice of nursing students is related to academic burnout. In agreement with our finding, a previous study showed that clinical practice stress is a significant factor influencing academic burnout [21]. Furthermore, positive professional self-concept reduced academic burnout [34]. Therefore, nursing instructors in schools as well as professionals in clinical practice environment must function as role models to improve satisfaction with instructors and meanings to the nursing profession. In addition to the results of our study showing differences in academic burnout according to clinical practice experience, another previous study reported that 49.2% of nursing students with clinical practice experiences wished to change their major [35]. Altogether, these finding suggest the need for in-depth reflection on nursing education.

Here, we observed different factors affecting academic burnout according to clinical practice experience. This suggests that interventions for prevention of academic burnout of students must be differentiated according to clinical experience of nursing students. Anxiety and depression were main factors affecting academic burnout of nursing students without clinical practice experience. In agreement with our findings, previous studies showed that nursing students feel anxious about adapting to university life and completing the curriculum [36] and that more than 50% of first- and second-year nursing students experience depression and require clinical treatment [37]. These evidence indicate the need for active interventions to prevent depression and anxiety such as in-depth guidance of professors on first and second year students' adaptation to university life, mentoring related to career and university life adjustment, and access to counseling programs [22, 25–31].

The main factors affecting academic burnout of participants with clinical practice experience were stress and major satisfaction. This finding is similar to that of previous studies, in which nursing students experience difficulties due to unfamiliar environments, changes in daily life, role ambiguity, and psychological atrophy during clinical practice [38, 39]. Additionally, consistent with our findings, negative experiences of clinical practice were correlated with academic burnout of nursing students [21, 40]. Therefore, programs such as simulation education, peer mentoring, and mindfulness programs to relieve stress of nursing students must be provided [41]. Moreover, as major satisfaction affects not only academic burnout, but also improvement of nursing competency [42] and is related to formation of nursing professional value [43], efforts must be made to improve major satisfaction, in addition to relieving stress of nursing students. Major satisfaction is related to various learning factors such as lecture rooms, clinical practice education, instructors, learning plans, and programs [44]. Therefore, such factors must be considered for interventions to improve major satisfaction.

Several limitations must be considered when interpreting this study's findings. The data was only collected from female nursing students; thus, differences in academic burnout according to gender could not be investigated. Additionally, other factors such as self-efficacy and nursing professional values that are related to academic burnout were not included in analysis of factors affecting academic burnout. However, this study provided evidence for strategies to reduce academic burnout by evaluating the level of academic burnout and factors affecting nursing students according to clinical practice experience. Moreover, this study is meaningful in that it presented directions of education and intervention required before and after clinical practice.

## Conclusion and suggestions

In this study, academic burnout of nursing students was correlated with stress, anxiety, and depression. In those students with no clinical practice experience, anxiety and depression had effects on academic burnout. In contrast, in those with clinical practice experience, stress and major satisfaction were factors affecting academic burnout. Our findings suggest the importance of actively developing and implementing intervention programs to reduce the academic burnout of nursing students. To enhance major satisfaction and meanings of being a nurse in nursing students, clinical practice environment and curriculum must be re-evaluated and improved. Additionally, to reduce stress, depression and anxiety and increase major satisfaction of nursing students, universities must provide support for students and establish a cooperative system with clinical site institutions.

## Data Availability

The data that support the findings of this study are available on request from the Corresponding author. The data are not publicly available due to privacy or ethical restrictions.

## References

[1] Arbabisarjou A, Hashemi SM, Sharif MR, Haji Alizadeh K, Yarmohammadzadeh P, Feyzollahi Z (2015). The relationship between sleep quality and social intimacy, and academic burn-out in students of medical sciences. Glob J Health Sci.

[2] Lee MK, Lim SH (2017). Relationships among academic burnout, stress coping and college adjustment in nursing students. Asia Pac J Multimed Serv Converg Art Humanit Sociol.

[3] Canadas-De la Fuente GA, Vargas C, San Luis C, Garcia I, Canadas GR, Emilia I. Risk factors and prevalence of burnout syndrome in the nursing profession. Int J Stud Nurs. 2015;52(1):240–249. 10.1016/j.ijnurstu.2014.07.001 10.1016/j.ijnurstu.2014.07.001 25062805

[4] Schaufeli WB, Martinez IM, Pinto AM, Salanova M, Bakker AB (2002). Burnout and engagement in university students: a cross-national study. J Cross Cult Psychol.

[5] Ko CM (2015). Mediating effect of stress on relationship between emotional intelligence and burnout among nursing college students. J Korean Soc Sch Health.

[6] Kim GM, Cha S (2013). Influence of emotional awareness, emotional expressiveness, and ambivalence over emotional expressiveness on college student adjustment in freshman nursing students. J Korea Cont Assoc.

[7] Rudman A, Gustavsson JP (2012). Burnout during nursing education predicts lower occupational preparedness and future clinical performance: a longitudinal study. Int J Stud Nurs.

[8] Jang J, Bae S, Kim G (2019). Effects of academic relationships on academic burnout in health professions students. Korean Med Educ Rev.

[9] Lee EH (2019). Effect of resilience on academic burnout of nursing students. J Korea Acad-Ind cooperation Soc.

[10] Kim MH, Oh IS (2019). A study on the relationship between parental overprotection and college student burnout: the moderated mediating effect of ego-resiliency and friendship quality. J Educ Innov Res.

[11] Rios-Risquez MI, Garcia-Izquierdo M, Sabuco-Tebar LA, Carrillo-Garcia C, Martinez-Roche ME (2016). An exploratory study of the relationship between resilience, academic burnout and psychological health in nursing students. Contemp Nurs.

[12] Deary IJ, Watson R, Hogston R (2003). A longitudinal cohort study of burnout and attrition in nursing students. J Adv Nurs.

[13] Yang SK, Jung MR (2016). The influences of academic-burnout, self-efficacy, major satisfaction on nursing professionalism in nursing students. J Learner-Centered Curric Instr.

[14] Cheung T, Wong SY, Wong KY (2016). Depression, anxiety and symptoms of stress among baccalaureate nursing students in Hong Kong: a cross-sectional study. Int J Environ Res Public Health.

[15] Chernomas WM, Shapiro C (2013). Stress, depression, and anxiety among undergraduate nursing students. Int J Nurs Educ Scholarsh.

[16] Hwang EH, Kim KH, Shin SJ (2016). The effect of life stress, sleep quality, and depression on suicidal ideation among nursing students. J Wellness.

[17] Lee E (2019). Effects of depression, anxiety, quality of sleep on excessive daytime sleepiness in nursing students. J Korea Acad-Ind Cooperation Soc.

[18] Ling L, Qin S, Shen LF (2014). An investigation about learning burnout in medical college students and its influencing factors. Int J Nurs Sci.

[19] Shin S, Yang EB, Hwang E, Kim K, Kim Y, Jung D (2017). Current status and future direction of nursing education for clinical practice. Korean Med Educ Rev.

[20] Cho HH, Kang JM (2018). Effect of resilience, coping, and mental health on burnout of student nurses. Child Health Nurs Res.

[21] Shin S, Hwang E. The effects of clinical practice stress and resilience on nursing students’ academic burnout. Korean Med Educ Rev. 2020;22(2):115–21. 10.17496/kmer.2020.22.2.115.

[22] An M, Kang AY, Kim YA (2017). Comparison of academic engagement, academic burnout, stress, and social support by grade among undergraduate nursing students. J Korean Soc Sch Health.

[23] Choi YS, Lee E, Lee D (2018). Relationship of locus of control, difficulties in emotion regulation, and clinical practice stress. J Korea Cont Assoc.

[24] Lee H (2016). The influencing factors of optimism and emotional Intelligence on depression among undergraduate students. J Korea Acad-Ind Cooperation Soc.

[25] Jang SJ (2020). Stress Standardization of Health Statistics Collection and Measurement.

[26] Radloff LS (1977). The CES-D scale: a self-report depression scale for research in the general population. Appl Psychol Meas.

[27] Chen KK, Choi SJ, Yang BC (2001). Integrated adaptation of CES-D in Korea. Korean J Health Psychol Soc.

[28] Spielberger CD, Speilberger CD, Sarason IG (1975). Anxiety: state-trait process. Stress and Anxiety.

[29] Kim JT, Shin DK (1978). A study based on the standardization of the STAI for Korea. New Med J.

[30] Shin H, Puig A, Lee J, Lee JH, Lee SM (2011). Cultural validation of the maslach burnout inventory for Korean students. Asia Pac Educ Rev.

[31] Park JA (2020). Factors influencing burnout in lower grades nursing student. J Learner-Centered Curric Instr.

[32] Jung SY (2019). A comparative study of college adjustment and life stress of nursing students by grades. J Ind Converg.

[33] Moon MY (2018). Relationship between academic stress, perceived health status, ego resilience and health promoting behavior by grade of nursing college students. Asia Pac J Multimed Serv Converg Art Humanit Sociol.

[34] Wang M, Guan H, Li Y, Xing C, Rui B (2019). Academic burnout and professional self-concept of nursing students: a cross-sectional study. Nurs Educ Today.

[35] Hwang E, Shin S (2018). Characteristics of nursing students with high levels of academic resilience: a cross-sectional study. Nurs Educ Today.

[36] Hughes M, Kenmir A, Innis J, O’Connell J, Henry K. Exploring the transitional experience of first-year undergraduate nursing students. J Nurs Educ. 2020;59(5):263–8. 10.3928/01484834-20200422-05. 10.3928/01484834-20200422-05 32352540

[37] Lee E (2019). Factors affecting depression in junior nursing students. J Digit Converg.

[38] Song H, Lim S (2019). A phenomenological study on the first clinical practice experience of nursing students. Asia Pac J Multimed Serv Converg Art Humanit Sociol.

[39] Park HJ, An HJ. Nursing student’s experiences on nunchi in clinical practice. J Korean Acad Soc Nurs Educ. 2019;25(1):48–57. 10.5977/jkasne.2019.25.1.48.

[40] Kim EK, Yu J, Lee JL (2019). The effect of clinical practice transitional shock and resilience on academic burnout of nursing students. J Learner-Centered Curric Instr.

[41] McCarthy B, Trace A, O’Donovan M, et al. Nursing and midwifery students’ stress and coping during their undergraduate education programmes: an integrative review. Nurse Educ Today. 2018;61:197–209. 10.1016/j.nedt.2017.11.029. 10.1016/j.nedt.2017.11.029 29227889

[42] Kim EJ, Lee OS (2021). A comparative study nursing competency and major satisfaction between nursing college students before and after their first clinical practice. J Digit Converg.

[43] Choi HJ (2017). Moderating and mediating effects of nursing professionalism between major satisfaction and college life adaptation among nursing students. J Korea Acad-Ind cooperation Soc.

[44] Guerra-Martin MD, Cano-Orihuela A, Martos-Garcia R, Ponce-Blandon JA (2021). Translation and first pilot validation study of the “Undergraduate Nursing Student Academic Satisfaction Scale” questionnaire to the Spanish context. Int J Environ Res Public Health.

